# Genotyping‐by‐sequencing approaches to characterize crop genomes: choosing the right tool for the right application

**DOI:** 10.1111/pbi.12645

**Published:** 2017-01-24

**Authors:** Armin Scheben, Jacqueline Batley, David Edwards

**Affiliations:** ^1^ School of Plant Biology and Institute of AgricultureUniversity of Western AustraliaPerthWAAustralia

**Keywords:** Breeding, Genomics, genotyping‐by‐sequencing, reduced‐representation sequencing, whole‐genome resequencing

## Abstract

In the last decade, the revolution in sequencing technologies has deeply impacted crop genotyping practice. New methods allowing rapid, high‐throughput genotyping of entire crop populations have proliferated and opened the door to wider use of molecular tools in plant breeding. These new genotyping‐by‐sequencing (GBS) methods include over a dozen reduced‐representation sequencing (RRS) approaches and at least four whole‐genome resequencing (WGR) approaches. The diversity of methods available, each often producing different types of data at different cost, can make selection of the best‐suited method seem a daunting task. We review the most common genotyping methods used today and compare their suitability for linkage mapping, genomewide association studies (GWAS), marker‐assisted and genomic selection and genome assembly and improvement in crops with various genome sizes and complexity. Furthermore, we give an outline of bioinformatics tools for analysis of genotyping data. WGR is well suited to genotyping biparental cross populations with complex, small‐ to moderate‐sized genomes and provides the lowest cost per marker data point. RRS approaches differ in their suitability for various tasks, but demonstrate similar costs per marker data point. These approaches are generally better suited for *de novo* applications and more cost‐effective when genotyping populations with large genomes or high heterozygosity. We expect that although RRS approaches will remain the most cost‐effective for some time, WGR will become more widespread for crop genotyping as sequencing costs continue to decrease.

## Introduction

The study of DNA polymorphisms forms the basis of modern genetics. By analysing the genomic variation between individuals and populations, polymorphisms can be used to identify genotypes, and connect them to phenotypes. Since the advent of high‐throughput sequencing technologies, the abundant and heritable single nucleotide polymorphisms (SNPs) have emerged as the most widely used genotyping markers. These can be used for linkage mapping, analysis of quantitative trait loci (QTL), association studies, marker‐assisted selection (MAS) and genomic selection (GS) in crops. Moreover, their generally low mutation rate means they can be used for genetic diagnostics and germplasm identification. The versatility of SNPs has also led to their widespread use in phylogenetics and phylogeography (McCormack *et al*., 2013). A major advantage of the single‐base resolution of SNPs is that it allows better detection of ‘perfect’ markers, which are causally linked to agronomic traits. A widely used range of methods for detecting SNPs using high‐throughput sequencing is known as genotyping‐by‐sequencing (GBS) (Andrews *et al*., 2016; Deschamps *et al*., 2012; He *et al*., 2014; Poland and Rife, 2012; Voss‐Fels and Snowdon, 2016). In comparison with earlier more complex and costly genotyping approaches such as those based on restriction fragment length polymorphism (RFLP) and simple sequence repeats (SSR), GBS can provide higher quantities of informative data by orders of magnitude. Although commercial SNP arrays still provide greater marker densities and are easier to analyse, they can be substantially more costly than GBS (Bajgain *et al*., 2016). Here, we review common GBS approaches and software tools to help researchers decide which approach best suits their research goals and to provide a perspective on future developments in plant genotyping.

## Reduced‐representation sequencing and whole‐genome resequencing approaches

GBS is now fuelling the transition from population genetics to population genomics, allowing high‐throughput identification of markers in crop populations at low costs (Voss‐Fels and Snowdon, 2016). Many important crops have been investigated using GBS to aid breeding endeavours, for example chickpea (Kujur *et al*., 2015), canola (Bayer *et al*., 2015; Bus *et al*., 2012), maize (Elshire *et al*., 2011; Gore *et al*., 2009), potato (Uitdewilligen *et al*., 2013), rice (Huang *et al*., 2009; Spindel *et al*., 2015), sorghum (Morris *et al*., 2013) and wheat (Poland *et al*., 2012b). Combined with phenotypic data, GBS approaches provide a powerful basis for rapid mapping and identification of genes underlying agronomic traits, which can then be introgressed into crop germplasm (Abe *et al*., 2012; Edwards *et al*., 2013).

Although GBS was initially developed as a reduced‐representation sequencing (RRS) approach using restriction enzymes to decrease genome complexity before sequencing (Baird *et al*., 2008; Miller *et al*., 2007), whole‐genome resequencing (WGR) approaches were soon applied to allow higher genomic resolution (Huang *et al*., 2009). Since the inception of GBS, it has undergone continuous development, giving rise to at least 13 approaches based on RRS and four on WGR (Table 1). Both RRS and WGR approaches profit from prior genomic information, although it is a prerequisite only for some WGR methods. This relative independence from prior genomic information means that RRS shows particular promise for characterizing the genomes of nonmodel species and previously neglected crops. Nevertheless, the increasing availability of crop genomes and further genetic resources indicates that whole‐genome approaches with high resolution may provide viable alternatives to RRS methods, particularly in plant breeding. Indeed, the reducing cost and changing type of NGS data being produced may cause a shift in the sequencing methods used by researchers towards more WGR approaches.

**Table 1:** Genotyping‐by‐sequencing methods currently available, divided into reduced‐representation sequencing (RRS) and whole‐genome resequencing (WGR) methods

RRS Methods	References
Restriction site‐associated DNA sequencing (RADseq)	Baird *et al*. (2008)
Elshire genotyping‐by‐sequencing (Elshire GBS)	Elshire *et al*. (2011)
Two‐enzyme GBS	Poland *et al*. (2012b)
Double‐digest RAD sequencing (ddRAD)	Peterson *et al*. (2012)
Sequence‐based genotyping (SBG)	Truong *et al*. (2012)
ezRAD	Toonen *et al*. (2013)
Restriction fragment sequencing (RESTseq)	Stolle and Moritz (2013)
Specific length amplified fragment sequencing (SLAF‐Seq)	Sun *et al*. (2013)
2bRAD	Wang *et al*. (2012)
Multiplexed shotgun genotyping (MSG)	Andolfatto *et al*. (2011)
Reduced‐representation library (RRL)	Van Tassell *et al*. (2008)
Complexity reduction of polymorphic sequences (CRoPS^™^)	Van Orsouw *et al*. (2007)
RAD Capture (Rapture)	Ali *et al*. (2016)
WGR Methods	
Sliding window WGR	Huang *et al*. (2009)
Parental inference WGR	Xie *et al*. (2010)
Parental inference WGR with individualized model	Rowan *et al*. (2015)
Skim genotyping‐by‐sequencing (SkimGBS)	Bayer *et al*. (2015)

### RNA sequencing and exome sequencing

RNA sequencing (RNA‐seq) and exome sequencing represent important alternative reduced‐representation approaches. Both of these approaches allow more selective sequencing, enabling focus on protein‐coding regions. Although coding sequences may amount to only 1%–2% of the genome (Pennisi, 2001), these sequences are likely to contain a high number of functional variants (Li *et al*., 2012) and a low number of repetitive regions. RNA‐seq generally uses direct sequencing of complementary DNA (cDNA) derived from RNA to obtain transcriptome sequences and quantify RNA transcript levels (Wang *et al*., 2009). While RNA‐seq is commonly applied for gene expression analyses, it is also a useful genotyping tool (Haseneyer *et al*., 2011). It has been used successfully for SNP genotyping in a number of crops. To help improve alfalfa cell wall composition, a total of 10 826 SNPs was detected using RNA‐seq of divergent cultivars (Yang *et al*., 2011). In a panel of diverse maize germplasm, 351 710 SNPs covering 22 830 annotated genes were identified with RNA‐seq (Hansey *et al*., 2012). Furthermore, based on SNP discovery in 27 000 wheat genes analysed with RNA‐seq, Ramirez‐Gonzalez *et al*. (2015) found markers for *Yr15*, a major disease resistance gene for wheat yellow rust.

An important advantage of RNA‐seq is that no prior genomic information is required (Wang *et al*., 2009). A further advantage of RNA‐seq is that the data generated for genotyping can also be used for expression analysis, shedding more light on the functional context of SNPs. However, RNA‐seq is limited by the bias in transcript abundance caused by the dependence of expression on tissue and time. Variants may go undetected because transcripts were not present in the sample while others may be overrepresented, leading to an increase of cost and effort if a more complete picture of the genotype is required. Additionally, RNA fragmentation during library preparation can introduce multiple further biases (Wang *et al*., 2009). RNA‐seq also requires high‐quality samples, which need to be processed rapidly because of the fast degradation of mRNA. Finally, the number of SNPs in coding regions is lower than in noncoding regions, so the variants obtained from RNA‐seq data may not be sufficient for GWAS, particularly in crops with high linkage disequilibrium.

The exome is the collection of all exons of protein‐coding genes in the genome. In a generalized exome sequencing workflow, genomic DNA is fragmented and probes are used to selectively hybridize to known target regions. Next, the probes bind to magnetic streptavidin beads in solution or microarrays, and the nontargeted DNA fragments are washed away. The bound DNA is subsequently enriched using PCR and then sequenced. Exome sequencing kits have been designed for numerous crops including wheat (Winfield *et al*., 2012) and barley (Mascher *et al*., 2013a). The wheat exome kit has allowed for discovery of previously unidentified markers in the genome which can be used in future genetic studies and marker‐assisted selection (Allen *et al*., 2013). Additionally, the barley kit has since been used to identify a mutation in the gene *HvPHYTOCHROME C* which is involved in flowering time, an important agronomic trait (Pankin *et al*., 2014). Exome sequencing has also been used to analyse variation in 94 eucalyptus genes related to wood properties, identifying 5905 SNPs (Dasgupta *et al*., 2015). Finally, exome sequencing has helped detect 1 395 501 SNPs in switchgrass (Evans *et al*., 2014), 97 075 SNPs in *Picea mariana* (Pavy *et al*., 2016) and 129 156 sequence variants in potato (Uitdewilligen *et al*., 2013).

Although exome sequencing does not allow analysis of gene expression levels, it enables sequencing of unexpressed alleles and genes that would not be found with RNA‐seq. In further contrast to RNA‐seq, targeted capture is scalable and can capture dozens to many thousands of genes. However, exome sequencing approaches rely on the existence of high‐quality reference genomes with accurate annotation. Low‐quality annotation of genomes can lead to variants being missed because probes are not designed for all relevant sites. Moreover, exome sequencing costs more than other RRS approaches such as RNA‐seq or GBS. In a US sequencing centre at internal prices for human samples including data analysis, exome sequencing can cost approximately $1000 per sample, while RNA‐seq can cost approximately $600 per sample (https://systemsbiology.columbia.edu/genome-center). Although exome sequencing is thus substantially more expensive than RNA‐seq, costs vary widely depending on sequencing coverage, choice of library preparation, location and sequencing provider. Compared to GBS, RNA‐seq and exome sequencing have the advantage that most transcripts and exons can be annotated using existing databases, which provides a functional context for SNPs. Most SNPs yielded by GBS, on the other hand, lie outside coding regions and are not easily annotated. Nevertheless, the many biases in RNA‐seq and the high cost and prior requirements of exome sequencing are the reasons that GBS, and particularly RRS, has become an increasingly popular genotyping method in diverse fields of biology (Andrews *et al*., 2016).

### Restriction site‐associated DNA sequencing

RRS methods generally employ restriction enzymes to digest genomic DNA in an initial step, but can differ in several ways including the number and type of enzymes used. The restriction enzyme‐associated DNA sequencing method (RADseq) follows a six‐step protocol (Baird *et al*., 2008; Miller *et al*., 2007). First, genomic DNA is digested with a single restriction enzyme. For sequencing of multiple samples in a single lane (multiplexing), adapters with barcodes are then ligated onto digested ends. After a sonication step, an adapter is ligated to the randomly sheared end. In the final steps, the library is size‐selected and RAD fragments with both adapters are PCR‐amplified. Elshire *et al*. (2011) simplified the RADseq protocol to four steps by implementing digestion and adapter ligation in a single well and eliminating random shearing and size selection steps, in an approach referred to here as Elshire GBS. With the Elshire GBS technique, barcoded adapters and common adapters with an overhang matching the restriction site are ligated onto digested fragments in a single sticky‐end ligation. Another important step in the diversification of RAD methods was the introduction of two enzymes in the double‐digest RAD protocol (ddRAD) (Peterson *et al*., 2012). Combining a low‐frequency and high‐frequency cutter to digest DNA, a barcoded adapter is ligated to one and a common adapter to the other restriction site. Samples are then pooled and size‐selected. Lastly, PCR is used to enrich the library and also to introduce a second barcode in the form of an Illumina index, increasing multiplexing potential. Similar to this approach is a modification of Elshire GBS known as two‐enzyme GBS (Poland *et al*., 2012b). The average coverage typically varies between these RRS methods. For instance, while RADseq and ddRAD involve sequencing fragments to moderate coverage between 5× and 15× (Fountain *et al*., 2016), Elshire and two‐enzyme GBS studies tend to reach low coverage ~1× (Swarts *et al*., 2014). Costs also differ by method, but vary from country to country and can undergo rapid changes. A rough estimate of the cost of Elshire and two‐enzyme GBS is <0.001$ per marker data point or around $30 per sample (De Donato *et al*., 2013; Poland and Rife, 2012), which is on a par with the cost of ddRAD and RADseq (Davey *et al*., 2011; Peterson *et al*., 2012). There are further less commonly used RRS methods, which differ from the above through the use of proprietary kits for adaptor ligation (ezRAD; Toonen *et al*. (2013)). New approaches for cheaper, more effective and universal RRS genotyping are constantly being developed (e.g., Ali *et al*., 2016).

The three main pitfalls of RRS are allele dropout, PCR duplicates and variance in coverage. In allele dropout, polymorphisms in the restriction enzyme recognition site prevent cutting and can thus lead to erroneous genotyping (Davey *et al*., 2013). Similar genotyping problems can be caused by stochastic uneven PCR duplication during library preparation, which can lead to biases towards certain alleles, although this does not affect the PCR‐free ezRAD. Finally, variance in coverage between loci can be caused by an amplification bias towards fragments of shorter length and with higher GC content. Beyond these common errors, the frequent use of methylation‐sensitive enzymes in RRS introduces an ascertainment bias. Single‐enzyme RRS using methylation‐sensitive enzymes such as PstI biases the sampling against intergenic regions, which can harbour almost half of trait‐associated SNPs (Hindorff *et al*., 2009).

### Whole‐genome resequencing

WGR differs from RRS in the lack of complexity reduction steps before sequencing. In a WGR approach known as skim genotyping‐by‐sequencing (SkimGBS), SNPs and genotypes are called using low‐coverage genomic reads, typically <1×, to make genotyping large populations viable (Bayer *et al*., 2015). This low coverage is common to WGR approaches and is sufficient for genomic analyses in recombinant populations with high‐quality parental genome sequences (Golicz *et al*., 2015). To simplify data analysis, heterozygous alleles are often eliminated by sequencing recombinant inbred line (RIL) or double‐haploid (DH) populations. The parental genomes and a reference sequence are often required for these mapping populations (Huang *et al*., 2009), although they can also be inferred using hidden Markov models (Xie *et al*., 2010), reducing the cost for deep sequencing of the two parents. Training the model on each individual sample refines this approach by allowing for variation in error rates (Rowan *et al*., 2015). This method is particularly useful in genotyping a constructed cross population, in which the parental lines are not known and parental genome sequences are not yet determined. As with RRS, the costs of WGR per marker data point change rapidly, vary with laboratory location and depend on the organism sequenced. However, a rough estimate of the costs of WGR is <0.0001$ per marker data point or approximately $80 per sample, based on the sequencing costs in Davey *et al*. (2011) and the number of SNPs found at 1.3× coverage in canola by Bayer *et al*. (2015). These costs per marker data point are an order of magnitude below those of RRS methods; however, the cost per sample will mostly remain higher for WGR, depending on the target coverage. Particularly in plants with large, polyploid genomes such as wheat, routine genotyping of populations with WGR is not financially feasible. While WGR is therefore still cost‐prohibitive for smaller laboratories and large genomes, it also benefits from being mostly unaffected by the biases of RRS (Table 2).

**Table 2:** Comparison of genotyping approaches

	Cost per sample^a^	Cost per marker data point^a^	SNP discovery rate	Analysis complexity	Prior genomic knowledge	Preferred population type	Drawbacks	Applications
RADseq	Low	Moderate	Low to moderate	Moderate	No	All	Labour‐intensive library preparation; high read depth variation	*De novo* SNP discovery, genome improvement, genetic mapping
Elshire GBS	Low	Moderate	Low	Moderate	No	All	High levels of missing data	*De novo* SNP discovery in simple genomes, genome improvement, genetic mapping
ddRAD	Low	Moderate	Low to moderate	Moderate	No	All	Sensitive to allele dropout; high‐quality sample required	*De novo* SNP discovery, genome improvement, genetic mapping
Parental inference WGR	High	Low	High	High	No	Biparental cross	High cost; inference is error‐prone	*De novo* SNP discovery, high‐resolution mapping of (complex) plant genomes, genome improvement
SkimGBS	High	Low	High	High	Yes	Biparental cross	High cost; need for prior genomic information	SNP discovery and high‐resolution mapping of (complex) plant genomes, genome improvement
SNP array	Moderate	High	High	Low	Yes	All	Ascertainment bias; need for prior genomic information	SNP discovery and high‐resolution mapping, genetic mapping
Exome sequencing	Moderate	High	Low	Moderate	Yes	All	Need for prior genomic information	SNP discovery in complex genomes, genetic mapping
RNA‐seq	Moderate	High	Low	Moderate	No	All	Biases in transcript abundances	SNP discovery in complex genomes, genetic mapping, expression analysis

a Relative costs shown may vary with factors including sample number and target coverage.

### The advance of long‐read sequencing

Although per‐base sequencing costs have plummeted during the last decade, second‐generation sequencing remains limited by short read length (~300 bases). Long‐read sequencing platforms such as Oxford Nanopore technologies and Pacific Biosciences single‐molecule real‐time sequencing can achieve reads >10 kb. Observed error rates on both platforms, however, have been higher than in conventional short‐read sequencing at ~15% (Jain *et al*., 2015; Korlach, 2013). Despite these high error rates, long‐read data have been used successfully for full‐length *de novo* assemblies of microbial (Goodwin *et al*., 2015; Loman *et al*., 2015; Quick *et al*., 2014), plant and human genomes (Berlin *et al*., 2015). The availability of long reads is important when assembling genomes because they allow improved locus identification and discrimination of paralogous or repetitive sequence by anchoring these within uniquely occurring parts of the genome. Sequencing chemistry is expected to develop further, improving long‐read sequencing accuracy (Jain *et al*., 2015). Already, hybrid approaches combining short‐read and long‐read data are proving viable (Koren *et al*., 2012; Madoui *et al*., 2015), although long‐read sequencing is still often cost‐prohibitive. While long‐read sequencing is unlikely to be cost‐effective for genotyping studies in the near future, improvements in existing reference genomes will benefit genotyping. As the costs of long‐read sequencing decrease in the future, together with an increase in read quality, we can expect genotyping methods to make more use of this technology.

## Applications of GBS

### Linkage and QTL mapping

Genetic linkage maps show the relative distances between markers along the chromosomes as determined by their recombination frequency. Such maps are important in breeding programmes as they facilitate QTL and association analysis. These analyses are powerful tools to identify genetic loci governing traits of interest using the principle of genetic linkage (Collard *et al*., 2005; Mohan *et al*., 1997).

RRS methods have been widely used in genetic mapping studies. RADseq was used to develop linkage maps and conduct QTL analysis in crops including aubergine (Barchi *et al*., 2011, 2012), barley (Chutimanitsakun *et al*., 2011) and cultivated strawberry (Davik *et al*., 2015). The GBS method was originally tested with 276 RILs from a maize mapping population, which led to the identification of 200 000 markers (Elshire *et al*., 2011). This method has seen wide use for linkage mapping and QTL analysis in diverse crops including rice (Spindel *et al*., 2013, 2015) and sweet cherry (Guajardo *et al*., 2015). The ddRAD method has been employed, for instance, to genotype canola (Chen *et al*., 2013) and for genetic linkage mapping in cultivated peanut (Zhou *et al*., 2014) and kiwifruit (Scaglione *et al*., 2012). WGR with imputation of parental genotypes has also proven effective for QTL mapping of 241 rice RILs sequenced at ∼0.06× coverage. A total of 270 820 high‐quality SNPs were identified, and a genetic linkage map was constructed, which allowed the identification of grain weight QTL (Yu *et al*., 2011). Furthermore, genotyping‐by‐resequencing has been applied frequently in rice, yielding a total of 1 493 461 SNPs identified in 150 RIL sequenced at 0.02× coverage. Using recombination bins to construct a linkage map, it was then possible to identify 49 QTL, including four linked to plant height (Huang *et al*., 2009). In sorghum, the same approach for 244 RILs sequenced at ~0.07× coverage led to the discovery of 7.76 million high‐quality SNPs and, after map construction, several major QTL for heading date and plant height (Zou *et al*., 2012). An ultradense genetic linkage map of wheat was also made using WGR of 90 DH individuals at 1.4× coverage (Chapman *et al*., 2015). Finally, genotyping of chickpea and canola identified 511 624 SNPs and 794 837 postfiltering SNPs, respectively. Based on these SNPs, numerous crossovers and gene conversions in both species could be identified (Bayer *et al*., 2015).

Linkage mapping and QTL analysis is carried out using all GBS methods, but these methods differ in their results. The number of markers required for a well‐resolved linkage map with high detection power depends on various factors including the level of recombination. When recombination is low, large numbers of closely placed markers such as those generated by WGR may be partially redundant. Moreover, when linkage disequilibrium is high among markers within a genomic region, only one may be selected for the analysis. In these cases, RRS approaches will be more cost‐effective while also providing sufficient markers for high‐resolution mapping. This can be the case in biparental populations, where several thousand markers may be sufficient (Beissinger *et al*., 2013). Nevertheless, with RRS approaches the mapping resolution remains fixed by the density of restriction sites. For this reason, linkage and QTL mapping in diverse populations can benefit from WGR. Further, WGR can identify causative SNPs, which is hard to achieve with RRS methods. Within the RRS group of methods, RADseq, Elshire and two‐enzyme GBS have been used frequently for linkage mapping and QTL analysis, but have some drawbacks when compared to ddRAD. Unlike the other two methods, ddRAD uses two restriction enzymes, which allows greater reproducibility in the recovery of a specific subset of the genome after size selection and reduces the size of the subset sampled leading to greater coverage (Peterson *et al*., 2012). Using Elshire and two‐enzyme GBS can produce highly skewed coverage of genomic positions (Beissinger *et al*., 2013). Thus, ddRAD is considered to provide more effective SNP genotyping compared with RADseq or Elshire and two‐enzyme GBS (Peterson *et al*., 2012). Nevertheless, when prior genomic information such as a reference panel is available, the lower and more uneven coverage of Elshire and two‐enzyme GBS can be compensated for with imputation (Torkamaneh and Belzile, 2015).

When a low number of markers is needed or the genome size is large, RRS methods are often more cost‐efficient and should be preferred to WGR. In uncommon cases, when genomes are complex and large, transcriptome and exome sequencing may prove effective at overcoming genotyping difficulties, as recently shown in hexaploid wheat (Akhunov, 2016). Generally, WGR offers the greatest cost‐efficiency per marker data point, and is particularly useful when recombination is high and many markers are needed for a well‐resolved genetic map in a species with a small‐ to moderate‐sized genome. WGR has the added benefit of increasing the chances of finding causative SNPs or genes, which allows development of ‘perfect’ markers. In the light of the decreasing costs of sequencing and the high cost of candidate gene validation, the use of WGR to increase the resolution of mapping studies is likely to become more common in the future.

### Genomewide association studies

Genomewide association studies (GWAS) use ancestral recombination events to identify the genetic loci underlying traits at high resolution. By employing association panels consisting of diverse genotypes, GWAS is able to pinpoint candidate genes precisely when linkage disequilibrium is relatively low, overcoming the limitations of less exact methods such as QTL mapping. Although commercial SNP arrays have been widely used for GWAS in crops such as rice, maize and soybean (Hwang *et al*., 2014; Li *et al*., 2013; Zhao *et al*., 2011), GBS methods are increasingly contributing data for GWAS. This is advantageous because GBS produces raw sequence reads, which can be reused more easily by other researchers.

In the potential energy crop *Miscanthus sinensis*, more than 100 000 SNPs were identified using RRS, which were used for a GWAS to detect associations between genetic variants and phenotypic traits such as cell wall composition, biomass and plant height (Slavov *et al*., 2014). Using Elshire GBS, 14 loci were identified in sorghum for the inflorescence branch length trait (Morris *et al*., 2013), and in soybean, loci associated with resistance to fungal stem rot and oil and protein content could be detected with similar methods (Bastien *et al*., 2014; Sonah *et al*., 2013). In canola, ddRAD detected two loci significantly associated with oil content of seeds (Fu *et al*., 2016). Finally, GWAS was used to dissect agronomic traits in rice landraces using low‐coverage WGR data, identifying 80 loci for 14 agronomic traits (Huang *et al*., 2010) and 32 further loci associated with flowering time and with ten grain‐related traits (Huang *et al*., 2012). The same approach was used to carry out a GWAS in *Setaria italica* (foxtail millet) varieties, which detected 512 loci associated with 47 agronomic traits (Jia *et al*., 2013).

The use of high‐density SNP data is crucial for the genetic dissection of important phenotypes in crop plants with GWAS. For this reason, genotyping methods producing low or medium marker densities may not be adequately powerful to find QTL with both large and small effects. WGR provides high marker densities and is well suited for GWAS, although high levels of heterozygosity can make WGR cost‐prohibitive because of the increased coverage required. While RRS using methylation‐sensitive enzymes or targeted sequencing of coding regions would introduce bias to the GWAS, the use of two enzymes in ddRAD or similar approaches would generate a more evenly sampled genomewide panel of markers (Peterson *et al*., 2012). Sampling noncoding regions is especially important as these may harbour the majority of trait‐associated markers (Hindorff *et al*., 2009). Alternatively to less biased RRS approaches, WGR can also achieve a sampling with low bias but at higher cost.

The structure of the genotyping population should be considered when selecting from the different GBS methods. Inbred lines derived from biparental crosses with the single‐seed descent or double‐haploid method are almost completely homozygous and thus better suited to WGR because coverage can be low and imputation is less problematic. Wild populations, on the other hand, may contain high degrees of heterozygosity. This means that higher coverage is needed to avoid missing too many heterozygous alleles, as imputation is not as accurate. Imputation of missing alleles plays an important role in increasing the cost‐efficiency of the low‐coverage sequencing data generated particularly by WGR and Elshire and two‐enzyme GBS (Andolfatto *et al*., 2011; Huang *et al*., 2015; Li *et al*., 2011). Researchers should thus consider the population structure and its effect on imputation when deciding which method to use. Low read depth in WGR and variation in read depth in Elshire and two‐enzyme GBS (Beissinger *et al*., 2013) can cause problems when genotyping heterozygotes, indicating that these methods are not well suited for the analysis of heterozygous populations. As with linkage mapping, genome size is a further important factor in deciding which genotyping method is best suited to GWAS. Sequencing a large population of a species with a large genome is best suited to RRS methods, which can achieve the required coverage without the substantial costs involved for WGR.

### Genome assembly validation and improvement

Many published genome assemblies remain works in progress because repetitive sequences and erroneous reads prevent accurate assembly. Assembly validation and improvement is therefore an important task. Usually, this is performed via the physical anchoring of genetically mapped markers. By linking the physical map (genome sequence) to the genetic linkage map, scaffolds can be anchored and ordered. In this way, linkage maps produced with high‐resolution GBS can be used to validate and fix assemblies (Mascher *et al*., 2013b).

For instance, placement of scaffolds in the recently published chickpea genome was validated with 5953 SNPs detected with RADseq. Mapped scaffolds containing SNPs enabled validation of scaffold structure based on the coherence of genotype calls, which allowed orientation of 75% of scaffolds (Varshney *et al*., 2013). RADseq also aided in assigning and ordering 83% of the genome of a *Heliconius* butterfly (Heliconius Genome Consortium, 2012). Similarly, two‐enzyme GBS has played an important role in anchoring the barley physical map to a genetic map (International Barley Genome Sequencing Consortium, 2012). More recently, Mascher *et al*. (2013b) used two‐enzyme GBS and WGR of barley to produce new genetic maps, which increased the amount of genetically anchored scaffolds of the genome by a factor of 3. Comparison of the two genotyping methods revealed similar mapping results. Lastly, *Actinidia chinensis* (kiwifruit) has been genotyped and mapped using ddRAD, which helped anchor an unmapped 120 Mbp and identify misjoined scaffolds (Scaglione *et al*., 2015).

The success of a given genotyping platform in validating and improving genome assemblies depends particularly on high marker density in the genetic maps. When a reference genome is available for validation and improvement and no *de novo* assembly is required, low‐coverage WGR and Elshire and two‐enzyme GBS approaches will be more efficient because they provide higher marker density and thus more precise anchoring. RNA sequencing and exome sequencing in particular may not provide sufficient evenly distributed markers as these approaches target the low‐diversity coding regions (Hansey *et al*., 2012). Nevertheless, when using WGR, Elshire GBS and two‐enzyme GBS approaches the availability of genomic resources for imputation is important to avoid high costs caused by the need for greater coverage. Genome size and complexity also affect the choice of genotyping approach for genome assembly validation and improvement. If enough resources are available for WGR at moderate coverage, this approach will yield the most markers. In large or highly repetitive genomes, however, RRS approaches will substantially lower costs while achieving adequate results.

### Marker‐assisted and genomic selection

Using genetic data to inform breeding efforts through MAS and GS is already prevalent in animal breeding, and has great potential to accelerate plant breeding while also improving its effectiveness (Varshney *et al*., 2014). MAS uses linkage disequilibrium (LD) between genetic markers and QTL to select plants with traits of interest for breeding programmes. This method has seen successful use for plant breeding in the public and private sector (Xu and Crouch, 2008), although the vast majority of publications on the subject are not considered to have real impact on breeding efforts (Collard and Mackill, 2008). While most MAS studies use SNPs, often low‐throughput Kompetitive Allele Specific PCR (KASP) and TaqMan assays are preferred to GBS because fewer markers are required. However, GBS is reported to play an increasing role in public and private breeding, for instance in tomato breeding (Foolad and Panthee, 2012). Different studies have also used RRS to identify markers useful for MAS such as those associated with *Lolium perenne* stem rust resistance (Pfender *et al*., 2011) and *Lupinus angustifolius* (lupin) stem blight resistance (Yang *et al*., 2013). Further, Yang *et al*. (2015) used WGR to detect and validate markers for MAS in commercial lupin cultivars, pointing to increased efforts to bridge the gap between publication and application.

In contrast to MAS, GS uses all genetic markers for a genotyped population to predict phenotypes. First, marker effects are estimated using a genotyped and phenotyped training population. This information is then used to generate a model which calculates genomic estimated breeding values (GEBVs) for the available genotypes. Finally, a breeding population can be created from selected individuals and used without further phenotyping (Meuwissen *et al*., 2001). In this way, GS substantially accelerates crop improvement, especially because of shorter generation times and the lack of phenotyping. An important advantage over MAS strategies is that GS can facilitate selection of complex traits controlled by many genes. Plant scientists have begun using GBS methods to conduct empirical GS studies, particularly in wheat. Poland *et al*. (2012a) and Rutkoski *et al*. (2014) applied two‐enzyme GBS to sets of elite wheat breeding lines and developed GS models with high prediction accuracies for yield and stem rust resistance, respectively. Genomic prediction based on GBS data has also been used in maize, where GBS performed as well as the more established SNP arrays and showed potential for harnessing variation for breeding populations (Crossa *et al*., 2013; Gorjanc *et al*., 2016).

Which GBS method is best suited to MAS and GS depends on the organisms and populations in question and on the budgetary constraints of the laboratory. Because codominant SNP markers can be used to develop PCR‐based markers, they are particularly useful for MAS. This is disadvantageous for enzyme‐based RRS, which also produces dominant markers when SNPs lie within the restriction site, although there are filtering methods to eliminate dominant markers. Marker density usually differs greatly between WGR and RRS methods and is crucial not only for QTL analysis but also for MAS and GS. In general, higher marker density increased the accuracy of predictive selection (Solberg *et al*., 2008). Particularly when LD is low, marker density needs to be high to maintain accuracy (Zhong *et al*., 2009). However, Hickey *et al*. (2014) use a simulation approach to estimate that an increase from 10 000 to 100 000 markers had little effect on accuracy. Their results suggest that substantially higher marker densities do not necessarily contribute to more accurate predictions, particularly when the number of phenotypes is low. In these cases, RRS methods may achieve high accuracy at lower cost than WGR.

## Bioinformatic tools for analysis and management of genotyping data

A bottleneck in large‐scale SNP genotyping is the analysis and management of vast amounts of genomic data. In the following, we outline widely used bioinformatics tools for GBS analysis pipelines and data management.

### Quality control

A preliminary step in the analysis of high‐throughput genotyping data is quality control of the raw sequences. High frequencies of low‐quality base calls and sequence contaminants, for instance, can impact downstream analyses by leading to higher computational demands and erroneous results. Tools such as FastQC (http://www.bioinformatics.bbsrc.ac.uk/projects/fastqc) produce summary reports, while others such as PRINSEQ (Schmieder and Edwards, 2011) are also capable of filtering and trimming reads. The FASTX‐Toolkit (http://hannonlab.cshl.edu/fastx_toolkit/) is a collection of command line tools providing quality reports and read trimming. A limitation of many quality controls tools is that quality assessments are produced individually for each sample and therefore require additional analysis for easy comparison. Recently developed tools such as MultiQC (Ewels *et al*., 2016) and Qualimap 2 (Okonechnikov *et al*., 2016) now enable multisample quality assessments. If low sequence quality or contamination is identified, Trimmomatic (Bolger *et al*., 2014) and AdapterRemoval 2 (Schubert *et al*., 2016) offer the highest throughput and high overall performance for removing contamination and low‐quality bases from single‐ and paired‐end FASTQ files.

### Short‐read mapping

Aligning the reads of all genotyped samples is a prerequisite for most downstream analyses such as variant calling. The majority of widespread high‐throughput sequencing platforms generate short sequence reads, which are aligned to reference genomes in a process known as short‐read mapping. When no reference sequence is available, it is necessary to generate a *de novo* assembly, a particularly challenging endeavour in plants (Birol *et al*., 2013; Schatz *et al*., 2012). Here, we will focus on short‐read mapping, as reference genomes for most major crops are available. Since the advent of high‐throughput short‐read sequencing, many alignment programs have been developed, often using the fast Burrows–Wheeler transform (BWT) approach, for example Bowtie/Bowtie2 (Langmead and Salzberg, 2012), BWA/BWA‐MEM (Li, 2013; Li and Durbin, 2009) and SOAP2 (Li *et al*., 2009c). BWA‐MEM is more accurate than Bowtie2 (Li, 2013), while Bowtie2 is more efficient at dealing with indels and paralogous sequences which are commonly found in plants (Langmead and Salzberg, 2012). While BWA/BWA‐MEM and Bowtie/Bowtie2 produce standard SAM output, the SOAP2 output is in a custom format and requires conversion. An advantage of SOAP2, however, is that it produces highly accurate alignments at the cost of mapping fewer reads than other software (Ruffalo *et al*., 2011). The high accuracy of SOAP2 makes it well suited for use in genotyping studies. Several software tools such as Stampy have also been developed for short‐read alignment using hash‐based approaches that are slower but more sensitive and can be combined with BWA to improve speed (Lunter and Goodson, 2011). Applied in‐depth comparisons and reviews of these and other tools have been published elsewhere (Fonseca *et al*., 2012; Li and Homer, 2010; Ruffalo *et al*., 2011; Trapnell and Salzberg, 2009) and indicate the performance of different algorithms can depend on the data used and the quality of the reference genome.

### Variant callers

Calling variants is essential for all genotyping analyses, and many tools have been developed for this purpose (Nielsen *et al*., 2011). The most widespread tools feature a probabilistic approach and include SOAPsnp (Li *et al*., 2009b), SAMtools (Li *et al*., 2009a), FreeBayes (Garrison and Marth, 2012), GATK (DePristo *et al*., 2011) and Platypus (Rimmer *et al*., 2014). However, the detection of SNPs in high‐throughput sequencing data still shows substantial conflict between different variant calling tools (Clevenger *et al*., 2015; O'Rawe *et al*., 2013; Pabinger *et al*., 2014). This inconsistency results partly from differences in how variants are identified. Some tools consider each site individually (SOAPsnp, SAMtools and GATK UnifiedGenotyper), while others assemble local haplotypes (GATK HaplotypeCaller, FreeBayes, Platypus). Both FreeBayes and GATK use Bayesian methods for modelling sequencing error, but SAMtools applies a hidden Markov mapping and assembly quality model to estimate error (Garrison and Marth, 2012; Liu *et al*., 2013). Although GATK is less flexible than other tools, requiring extensive formatting of input, it may offer increased accuracy by improving alignments locally before calling variants. GATK and FreeBayes also offer the advantage of allowing the user to select a ploidy level that is not restricted to haploid or diploid, which is useful as many crops are polyploid. Heuristic SNP callers such as VarScan2 (Koboldt *et al*., 2012) and SGSautoSNP (Lorenc *et al*., 2012) use information such as abundance and quality of data to help improve variant calling. Because they generally require more computational resources than probabilistic approaches, they are less commonly used. SGSautoSNP reduces sources of error introduced by reference‐based SNP discovery, as it identifies variants between the mapped reads of multiple samples.

Filtering SNPs for read depth, read mapping quality, base quality and minor allele frequency can be carried out by most variant calling tools. There are also several stand‐alone tools with extended filtering capabilities such as VCF tools (Danecek *et al*., 2011). Comparative studies of different variant calling tools have supported different tools as the most accurate and efficient (Clevenger *et al*., 2015; Liu *et al*., 2013; Pabinger *et al*., 2014). These different outcomes indicate that the result of variant calling with various tools may also depend on the data analysed. It is therefore difficult to pinpoint a generally superior tool. Rather, a consensus approach focusing on variants independently identified by different tools offers a solution to the conflict (Pabinger *et al*., 2014).

Analysis pipelines for read mapping and variant calling have also been developed specifically for GBS data. Common GBS analysis pipelines are TASSEL‐GBS (Glaubitz *et al*., 2014), Stacks (Catchen *et al*., 2011) and UNEAK (Lu *et al*., 2013). Compared to pipelines such Stacks and UNEAK, TASSEL‐GBS is specifically designed to handle large quantities of low‐coverage data. UNEAK and Stacks are better suited for *de novo* approaches in species without reference genomes. A recent comparison of GBS pipelines showed that, similarly to the stand‐alone variant calling tools, the variants found intersect broadly, but a moderate proportion remains inconsistent between pipelines (Torkamaneh *et al*., 2016). Major differences can be expected in pipelines as they may differ not only in variant calling algorithms and models but also in read mapping and processing (O'Rawe *et al*., 2013). Indeed, GBS pipelines increase user‐friendliness and ease of variant calling at the cost of flexibility and transparency of parameters. A consensus approach to cross‐validate variants is therefore also important for GBS pipelines.

### Analysing quantitative trait loci and carrying out association studies

The statistical analysis of genetic variants to find QTL and carry out GWAS has a mature well‐defined framework. The *de facto* standard tools are R/qtl (Broman *et al*., 2003) for QTL analysis and PLINK (Purcell *et al*., 2007) for GWAS. R/qtl provides various QTL mapping approaches and allows correction for covariates such as specific experimental treatments. The tool QTLNetwork (Yang *et al*., 2008) expanded on these capabilities by introducing more complex models to take into account subtle factors including interactions between QTL and the environment. Further tools commonly used for QTL analysis are MapQTL (Van Ooijen, 2004), QTL cartographer (Basten *et al*., 2004) and Mapmaker (Lander *et al*., 2009).

PLINK is a command line utility with various functions for analysis of variant data and built‐in diagnostic tools to assess quality. PLINK employs standard regression for GWAS. However, standard regression may not be sensitive enough when the frequency of the variant is low (Ma *et al*., 2013). Other tools such as Random Jungle (Schwarz *et al*., 2010) use fast random forest methods, which can be more sensitive than traditional statistical approaches. Further popular tools for GWAS also include TASSEL (Bradbury *et al*., 2007) and the R packages GenABEL (Aulchenko *et al*., 2007) and SNPassoc (Gonzalez *et al*., 2007).

### Annotation of variants

Variant annotation is important for connecting genetic variants such as SNPs with phenotypic effects. The annotation of variants aims to categorize the functional impact of variants on protein‐coding genes and regulatory regions. To enable annotation, an annotated reference genome or transcript set is required. As most annotation tools are optimized for human genomes, additional formatting of reference input is often required. Widely used variant annotation tools include Annovar (Wang *et al*., 2010), SnpEff (Cingolani *et al*., 2012), Variant Effect Predictor (VEP) (McLaren *et al*., 2010) and VariantAnnotation (Obenchain *et al*., 2014). The choice of reference genome or transcript set and of annotation software can have substantial impact on annotation results. In a comparison between VEP and Annovar, for instance, the consensus for high‐impact variants such as those causing loss‐of‐function was between 65% and 87% (McCarthy *et al*., 2014). Moreover, predictions of variant effects using common algorithms only found a consensus of 5% for high‐impact deleterious variants (Chun and Fay, 2009). A reason for this moderate‐to‐low concordance is that annotation tools define noncoding features differently. For instance, SnpEff uses 5 kb to define upstream and downstream regions, while Annovar uses 1 kb. Annotation tools also differ in their output format. Annovar produces a tab‐separated file, while SnpEff, VariantAnnotation and VEP produce extended VCF files with annotations included in the ‘INFO’ field. Unlike other annotation tools, SnpEff groups variants affecting the transcriptional unit into four categories based on the level of impact. Variant annotation with common tools is effective but not yet fully matured. Stringent filtering and consensus approaches are likely to increase their accuracy in the short term, while wider adoption of standard ontology terms and more refined treatment of the potentially functionally important noncoding regions (Alexander *et al*., 2010) will improve annotation in the long term.

### Management of genotyping data

Data management systems are essential to capture and manage the vast quantities of genomic data for applied breeding. However, storage and integration of the increasing amounts of data are a major challenge (Batley and Edwards, 2009; Lee *et al*., 2012). Sequencing instruments typically generate FASTQ files containing quality encoded sequencing reads. These files are usually 3–4 times larger than the aligned reads in the standard BAM format. Once variants have been identified, they are generally stored in the variant call format (VCF) (Danecek *et al*., 2011), which can be compressed with tabix (Li, 2011) to files about 3–5 times smaller. A major challenge is that potentially important information is often lost downstream in the analysis pipeline and during compression. For this reason, it is often not possible for researchers to delete raw FASTQ files or keep only compressed data. A solution to the storage of large‐scale genomic data may lie in the decreasing cost of cloud‐based storage systems such as those offered by Amazon Web Services (O'Driscoll *et al*., 2013).

Increasing data protection and efficient access are also important for the management of genotyping data. Data management systems such as iRODS (Rule‐Oriented Data management systems) can help simplify data replication and allow adjustable levels of metadata to enhance accessibility (Chiang *et al*., 2011). Finally, broader access to research communities can be provided with public crop databases hosting genotyping data such as those established for maize (maizeGDB; http://www.maizegdb.org), tomato (http://solgenomics.net) and wheat (wheatIS; http://wheatis.org). These community databases increase accessibility of research data and help drive community‐based storage and analysis solutions.

## Conclusion

GBS represents a powerful suite of genotyping approaches with wide‐ranging applications and the potential to accelerate plant breeding programmes. However, these approaches vary in their costs per marker data point, in the types of data produced and in errors and potential biases. When selecting a method, researchers must therefore consider genome size, population structure, prior genomic information available and their varying influence on the different methods. The choice of software tools should be made consciously with respect to the specific genome and the analytical goals. While RRS approaches remain cost‐effective, WGR methods, possibly in combination with long‐read sequencing, will increase in use as sequencing costs continue to drop.
